# Milk yield and grazing behavior are influenced by the amount of concentrate but not the pregrazing pasture height offered to late-lactation grazing dairy cows

**DOI:** 10.3168/jdsc.2025-0944

**Published:** 2026-05-20

**Authors:** Meaghan L. Douglas, Joanna W. Heard, Khageswor Giri, Greg L. Morris, Monique J. Berkhout, William J. Wales, Joseph L. Jacobs, Joanna E. Newton, Marlie M. Wright

**Affiliations:** 1 Agriculture Victoria Research, Department of Energy, Environment and Climate Action, Ellinbank, VIC 3821, Australia; 2 Agriculture Victoria Research, Department of Energy, Environment and Climate Action, Hamilton, VIC 3300, Australia; 3 AgriBio, Agriculture Victoria Research, Department of Energy, Environment and Climate Action, Bundoora, VIC 3086, Australia; 4 School of Applied Systems Biology, La Trobe University, Bundoora, VIC 3083, Australia

## Abstract

In temperate regions of the world, such as southeastern Australia, the majority of dairy farms are pasture-based, with supplementary concentrates fed to cows in the parlor during milking to varying degrees based on location, season, and stage of lactation. Therefore, there is considerable variation between farms in the amount of pasture and concentrate fed. This experiment aimed to investigate the milk production, DMI, and grazing behavior of dairy cows offered pasture at different pregrazing pasture heights coupled with different amounts of concentrate. Thirty-six Holstein-Friesian dairy cows in late lactation were used in the 25-d experiment, during which there were two 6-d measurement periods. Six cows were randomly allocated to each of the 6 treatment combinations using a completely randomized design, balanced for DIM, and both BW and milk production based on data from the 7 d before the beginning of the experiment. The treatment combinations consisted of offering cows one of 2 pasture heights (10 or 14 cm, measured to ground level) and one of 3 amounts of concentrate (3, 5, or 7 kg DM/d). Pasture height did not influence milk yield or composition, but cows offered a higher pregrazing pasture height had increased pasture DMI (estimated using the n-alkane technique). Cows offered 7 kg DM of concentrate/d produced the greatest yields of milk but averaged a 4 kg DM/cow per d lower pasture DMI compared with cows offered 3 or 5 kg DM of concentrate/d. There was no difference in total daily eating time between any of the treatment groups, whereas total daily ruminating time was lowest for cows offered 7 kg DM of concentrate/d compared with cows offered 3 or 5 kg DM of concentrate/d. As pasture is generally the lowest-cost feed source on pasture-based farms, substituting pasture consumption for concentrate can reduce profit while also increasing postgrazing residuals, which affects subsequent pasture regrowth. Balancing concentrate feeding and pasture utilization in grazing-based dairy systems is an important principle for pasture-based dairy farms in temperate regions of the world.

## Highlights

• Offering pasture of a greater pregrazing height increased pasture DM intake.
• Offering 7 kg DM of concentrate resulted in greater yields of milk, milk fat, and milk protein.
• Total daily eating time did not differ between any of the treatment groups.
• Offering 7 kg DM of concentrate reduced daily rumination time.

## Graphical Abstract

This experiment investigated the effects of offering different pregrazing pasture heights and concentrate amounts on milk production, individual cow dry matter (DM) intake, and grazing behavior of dairy cows. Understanding this can help farmers correctly allocate feed to their herds. Thirty-six Holstein-Friesian cows were assigned to 1 of 2 pregrazing pasture heights and 1 of 3 concentrate amounts, and monitored for 25 days, including two 6-day measurement periods. Pasture height did not influence milk yield or composition, whereas feeding 7 kg DM of concentrate did increase milk yield and yields of milk fat and protein compared with feeding 3 or 5 kg DM of concentrate. Cows offered pasture of a higher pregrazing height (14 cm) and 3 or 5 kg DM of concentrate had increased pasture DM intakes. Farmers need to consider the optimal amount of concentrate and pregrazing pasture height to offer their cows to improve milk production and ensure efficient pasture utilization.

**Figure:**
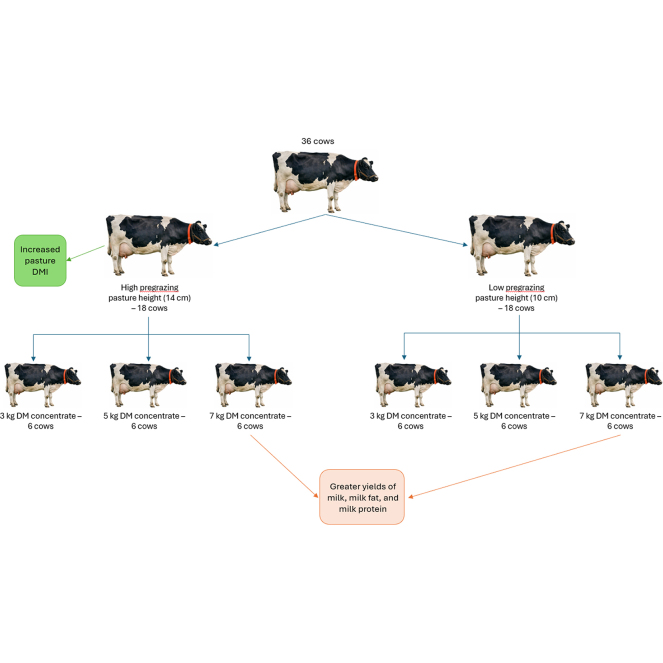

The dairy industry in temperate regions of the world, including Australia, Ireland, and New Zealand, is predominantly pasture based due to its inherent low cost. However, pasture alone does not support high (>30 kg milk/cow per d) milk production from dairy cows (Kolver and Muller, 1998), and many farmers supplement with purchased feeds, including concentrates fed in the parlor during milking. In pasture-based systems, DMI of pasture is seen as the first limiting factor for milk production, and therefore supplementary concentrates are often fed to increase milk production, fill feed deficits, and maintain cow condition (Kolver et al., 1998; Hills et al., 2015). There is potential for substitution, particularly when high amounts of concentrate are fed, which can reduce the amount of pasture consumed and properly utilized (Stockdale et al., 1997; Stockdale, 2000) and reduce grazing time (Bargo et al., 2002). Although feeding more concentrate can lead to higher milk production, care needs to be taken to ensure that it is not overfed, negatively affecting pasture DMI, pasture utilization, and subsequently profitability.

The amount of pasture offered to grazing dairy cows influences pasture DMI (Alvarez-Hess et al., 2021), as does pasture mass (the amount of above-ground pasture per unit area available to a grazing animal; Hodgson, 1979). Pérez-Prieto et al. (2011) found that cows grazing low-mass pastures (19 kg OM/cow per d) had reduced pasture DMI. Phillips and Hecheimi (1989) reported that cows offered a low pasture height (8 cm, to ground level) and no supplement had an increased grazing time, but this was not observed when cows were offered a high pasture height (12 cm, to ground level). Dry matter intake was not measured in that experiment, but it was acknowledged that DMI is greater at higher pasture availability. Feeding supplements to grazing dairy cows can affect pasture DMI, as cows generally substitute the supplement for some of the pasture that they would have otherwise consumed (Leaver et al., 1968; Stockdale et al., 1997). This can result in cows under-grazing the sward, which affects utilization and future growth of the pasture (Stockdale, 2000; Fulkerson et al., 2005). In the review by Poole et al. (2019), postgrazing residual increased by 1.4 mm for every kilogram DM of concentrate that was consumed, and for every 10 mm increase in the postgrazing residual, marginal milk response declined by 9%. As many dairy cows in temperate regions of the world are fed a mixture of pasture and concentrates, understanding the impacts of pregrazing pasture height and mass, and the amount of concentrate fed, in combination, is critical.

This experiment aimed to investigate the impacts of offering pasture to grazing cows at one of 2 pregrazing pasture heights and offering one of 3 amounts of concentrates on milk production, pasture DMI, and grazing behavior. A simultaneous aim was to provide data that could be used in the development of DMI prediction equations (J. W. Heard, K. Giri, M. L. Douglas, and M. M. Wright, unpublished). The hypotheses tested here were that (1) milk yield and pasture DMI would increase when pasture was offered at a higher pregrazing pasture height; (2) offering greater amounts of concentrate would increase milk yield but decrease pasture DMI; and (3) total daily eating time would be greater for cows offered lower pregrazing pasture height and reduced amounts of concentrate. Target pasture allowance was approximately 30 kg DM/cow per d, regardless of pasture height.

The experiment was conducted in May 2023 (autumn) at the Agriculture Victoria Research Ellinbank SmartFarm, Victoria, Australia (38°14′S, 145°56′E) with approval from the Department of Energy, Environment and Climate Action Agricultural Research and Extension Animal Ethics Committee (AEC approval number 2023–02).

Thirty-six spring-calving Holstein-Friesian dairy cows in late lactation were offered one of 2 pregrazing pasture heights (average 10 cm, termed low, **L**; or 14 cm, termed high, **H**; measured to ground level) and one of 3 concentrate amounts (3, 5, or 7 kg DM/cow per d). Six cows were randomly allocated to each of the 6 treatments (termed L3, L5, L7, H3, H5, and H7) using a completely randomized design. Each treatment group was balanced for cow age, DIM, as well as 7-d average BW and 7-d average milk production (determined in the 7 d before commencement of the experiment). Cows were in at least their second lactation, between 3 and 9 yr of age, and were in late lactation at an average of 274 DIM (range 218 to 303 DIM) at the beginning of the experiment.

The experiment was conducted over 25 d. During the adaptation period (the first 4 d) cows were adapted to both their allocated pasture height and concentrate amount. From the beginning of the 6-d dosing period (commencing on d 5), cows began to graze in plots in their allocated treatment groups, and the n-alkane technique was employed to estimate pasture DMI in individual cows. Each of the 3 groups in the L treatment were grazed adjacent to each other in one paddock, while each of the 3 groups on the H treatment were grazed adjacent to each other in a different paddock. To prepare the paddocks for the experiment, the rotation length was 38 and 55 d for the L and H treatment paddocks, respectively, to ensure a desired pregrazing pasture height. There were then two 6-d measurement periods (d 11 to 16, and d 19 to 24) separated by a 2-d “wash-out” period during which cows on the L pasture height swapped to the H pasture height, and vice versa, similar to a crossover design. Concentrate allocation remained unchanged, with n-alkane dosing continuing during the “wash-out” period. Cows swapped between pregrazing pasture height only during the “wash-out” period; it was deemed appropriate in length for cows to adapt as both the pasture allowance and pasture mass changed only to a minor degree.

Pasture height for each plot of pasture fed to each of the 6 treatment groups was measured by using a rising plate meter (Ellinbank Plate Meter, Jenquip, Feilding, New Zealand; Earle and McGowan, 1979), with 50 measurements made before cows entered each plot (pregrazing). Pasture height is presented here as compressed pasture height. All cows were allocated approximately 30 kg DM of pasture/cow per d (to ground level), split into 2 equal halves, with a fresh strip of pasture allocated after each milking. Postgrazing residuals of 1,500 kg DM/ha were targeted. Back-fencing was used to prevent cows from re-grazing the previous day's pasture. Pasture allowance was measured by calibrating a rising plate meter using quadrat cuts to construct regression equations that related DM/ha to plate meter readings of compressed pasture height. As cows on the L and H treatments grazed in separate paddocks at each measurement period (2 paddocks in each measurement period), quadrat cuts were taken from each of the paddocks, and one calibration equation was generated per paddock (4 calibration equations in total). To control pasture allowance, plot size was altered. Actual allowances achieved averaged 32.0 and 30.5 kg DM/cow per d to ground level for the L and H treatments, respectively. Actual postgrazing residuals averaged 1,630 and 1,460 kg DM/ha for the L and H treatments, respectively.

Pasture mass was calculated by using the pasture height measured by rising plate meter pre- and postgrazing for each of the 6 treatments during each day of the two 6-d measurement periods. Cows on the L pasture height treatment (10 cm) grazed in plots with an average (±SD) pregrazing pasture mass of 3,360 ± 340 kg DM/ha (measured to ground level), and consisted of the following: estimated ME, 10.8 ± 0.5 MJ/kg DM; CP, 17.4 ± 1.4% DM; NDF, 43.7% ± 2.1% DM; ADF, 27.9% ± 2.1% DM; crude fat, 5.1% ± 0.6% DM; WSC, 23.7% ± 1.8% DM; and NFC, 25.0% ± 2.0% DM. Meanwhile, cows on the H pasture height treatment (14 cm) grazed in plots with an average (±SD) pregrazing pasture mass of 3,670 ± 300 kg DM/ha (measured to ground level), and consisted of the following: estimated ME, 10.8 ± 0.4 MJ/kg DM; CP, 18.7% ± 1.5% DM; NDF, 43.8% ± 2.1% DM; ADF, 28.6% ± 1.4% DM; crude fat, 5.2% ± 0.6% DM; WSC, 19.7% ± 1.8% DM; and NFC, 20.8% ± 2.2% DM.

In addition to the pasture, cows were offered either 3, 5, or 7 kg DM of concentrate/cow per d. The concentrate mix (Reid Stockfeeds, Trafalgar, VIC, Australia) consisted of (% of DM offered) 23.0% crushed wheat, 43.5% crushed barley, 23.5% canola meal, and 8.2% minerals (mineral mix consisted of AMS Go Cow 200 [Animal Mineral Solutions, Shepparton, VIC, Australia], magnesium, limestone, salt, and sodium bicarbonate), and was fed twice daily in equal portions in the parlor during milking. Concentrate intake was calculated as the concentrate offered minus the concentrate refused. The concentrate mix offered was the same for all cows and consisted of the following: estimated ME, 13.1 ± 0.2 MJ/kg DM; CP, 17.4% ± 1.3% DM; NDF, 14.0% ± 1.1% DM; ADF, 7.3% ± 0.2% DM; starch, 41.7% ± 3.1% DM; WSC, 5.9% ± 0.7% DM; and NFC, 57.9% ± 3.0% DM.

Milk yield of each cow was recorded twice daily as per normal farm practice using a DeLaval Delpro milk metering system (DeLaval International, Tumba, Sweden), and milk composition was measured 3 times (p.m. + a.m.; 6 milkings in total) during each of the two 6-d measurement periods using the methodology described by Douglas et al. (2021). Energy-corrected milk yield standardized to 4.0% fat and 3.3% protein was calculated using the following formula (Tyrrell and Reid, 1965):

ECM (kg/cow per d) = milk yield (kg) × (376 × fat% + 209 × protein% + 948)/3,138.

Body weight of each cow was measured twice daily by walking cows over electronic scales (DeLaval Automatic Weight System AWS100, Tumba, Sweden) after each milking.

Individual cow DMI of pasture was estimated using the n-alkane technique developed by Mayes et al. (1986). All cows were dosed twice daily with gelatin capsules containing a known concentration of a synthetic n-alkane for 20 d, and fecal samples were collected from each cow twice daily during each of the two 6-d measurement periods using the methodology described by Wright et al. (2025). Representative samples of the pregrazing pasture offered were collected daily for each of the 6 treatments, as were representative samples of the concentrate offered to the cows. The pasture samples collected were representative of the height of the pasture on offer to the cows (to grazing height) and were collected using electric shears at several points along a transect for each treatment plot. These samples were processed as per Wright et al. (2025).

The concentrations of n-alkanes were analyzed at a commercial laboratory (AgriBio, Bundoora, Australia) by GC using the method described by Dove and Mayes (2006) and modified by Liu et al. (2022). The average daily individual pasture DMI (kg DM/cow per d) of each cow was estimated from the concentrations of the n-alkanes in feces, the concentrate mix and pasture, and the daily dose rate of C_32_. Average daily pasture DMI (kg DM/cow per d) was estimated using the following equation (Dove and Mayes, 1991):

$$\mathrm{intake}=\frac{\left(\frac{F_{i}}{F_{j\;}}\times \left(D_{j\;}+I_{c}\times C_{j}\right)-I_{c}\times C_{i}\right)}{\left(H_{i}-\frac{F_{i}}{F_{j}}\times H_{j}\right)},$$

where *F_i_* and *H_i_* represent the fecal and herbage n-alkane concentrations of the C_33_ alkane, *F_j_* and *H_j_* represent the fecal and herbage n-alkane concentrations of the C_32_ alkane, *D_j_* represents the daily dose rate of the C_32_ alkane, *I_c_* represents the known intake of the concentrate mix, *C_i_* represents the n-alkane concentrations of the C_33_ alkane in the concentrate mix, and *C_j_* represents the n-alkane concentrations of the C_32_ alkane in the concentrate mix.

Pasture samples for nutritive analysis were collected pre- and postgrazing (to ground level) using electric shears at several points along a transect of each plot for each of the 6 treatments during each day of the two 6-d measurement periods, and processed as per the methodology of Douglas et al. (2025). Samples were sent for analysis at a commercial laboratory (Dairy One Forage Laboratory, Ithaca, NY) for nutritive characteristics (CP, NDF, ADF, starch, NFC, lignin, ash, and calculated ME) by wet chemistry (AOAC International, 2000; Dairy One, 2020). A sample of the concentrate mix in each measurement period was also collected and analyzed by wet chemistry at a commercial laboratory (Dairy One Forage Laboratory, Ithaca, NY).

Grazing behavior was estimated for all cows during each of the two 6-d measurement periods using RumiWatch noseband sensors (Itin and Hoch GmbH, Liestal, Switzerland). The noseband sensors were used to measure eating, ruminating, and idling times, and the number of bites and chews. Data were analyzed using RumiWatch Manager 2 software (version 2.2.0.0) and RumiWatch Converter software (version 0.7.4.13; Itin and Hoch GmbH). Data were converted into hourly summaries and summed for each 24-h period (0600 h to 0600 h the following day) before statistical analysis.

Daily milk yield, milk composition, and pasture DMI data were averaged across each measurement period to obtain a mean value for each cow within each period. Feeding behavior data and activity durations were summed over each 24-h interval and then averaged across each measurement period for each cow. These cow-level mean values were analyzed using an analysis of covariance (**ANCOVA**) model. The ANCOVA model included the main effects of pasture height and concentrate level, and their interaction, as the treatment structure, with effect of cows and period within cow as the blocking structure. Pre-experimental measurements (age, DIM, BW, and average milk yield) were included as covariates in the ANCOVA model.

Model assumptions were checked by examining histograms of residuals and plots of residuals versus fitted values for normality and homogeneity of variance. Treatment effects were considered significant at *P*-values <0.05. Pairwise comparisons among treatment means were performed using Fisher's protected least significant difference test, and significant differences indicated using Duncan's letters. All statistical analyses were conducted using GenStat (version 23, VSN International Ltd., Hemel Hempstead, UK).

Pregrazing pasture height (L or H) did not influence milk yield or composition significantly. However, pasture DMI was greater (*P* = 0.010) for cows offered pasture of a higher pregrazing pasture height (Table 1). The 2 pregrazing pasture heights investigated in this experiment resulted in different pregrazing pasture masses; to control pasture allowance, plot size was altered for each treatment. Actual pasture allowances were 32.0 and 30.5 kg DM/cow per d for cows on the L and H treatments, respectively. There were, however, some similarities in pregrazing pasture mass; pasture mass was similar for cows on the L3, H3, H5, and H7 treatments, which may reflect the use of different calibration equations between treatments. Previous Australian research has reported an increase in pasture DMI as the available pasture mass increased from 3,100 to 4,900 kg DM/ha (Wales et al., 1999), but this decreased pasture utilization. Poole et al. (2019) reported a decline in pasture DMI with increasing supplement use (0.33 kg/kg of supplement), with a corresponding increase in postgrazing residual. In our experiment, postgrazing residual increased for cows offered 7 kg DM of concentrate per day, resulting in the lowest pasture utilization. These cows had the lowest pasture utilization, and a greater postgrazing residual. Pasture utilization was greater, on average, for cows grazing H compared with L pregrazing pasture height, regardless of concentrate amount (51% vs. 57%, respectively), which contrasts with previous research.

**Table 1:** Average DMI of concentrates and pasture, plus total intake (kg DM/cow per d) for cows in each of the treatments, and pre- and postgrazing pasture mass, averaged over the two 6-d measurement periods; pasture utilization (%) is also presented for the interaction treatments

Variable	Pasture height^1^		Concentrate amount^1^			Interaction (Int.)						SED^2^			*P*-value		
	L	H	3	5	7	L3	L5	L7	H3	H5	H7	Height	Concentrate	Int.	Height	Concentrate	Int.
Concentrate intake	4.8	4.8	2.9	4.8	6.7	2.9	4.8	6.7	2.9	4.8	6.7	—	—	—	—	—	—
Pasture intake	20.0^a^	21.3^b^	22.2^b^	21.8^b^	18.0^a^	21.9^c^	21.8^c^	16.4^a^	22.5^c^	21.8^c^	19.6^b^	0.48	0.60	0.34	0.010	<0.001	0.022
Total intake	24.8	26.1	25.1^a^	26.6^b^	24.7^a^	24.8^ab^	26.6^c^	23.1^a^	25.4^bc^	26.6^c^	26.3^bc^	0.48	0.60	0.84	0.010	0.007	0.023
Pregrazing pasture mass	3,360^a^	3,670^b^	3,570	3,530	3,440	3,600^bc^	3,340^ab^	3,130^a^	3,550^bc^	3,720^c^	3,750^c^	95.7	117.2	165.8	0.002	0.498	0.023
Postgrazing pasture mass	1,630^b^	1,460^a^	1,460	1,520	1,660	1,620	1,550	1,710	1,300	1,480	1,610	69.7	85.4	120.8	0.021	0.059	0.277
Pasture utilization^3^	—	—	—	—	—	55	54	45	63	60	57	—	—	—	—	—	—

a–c Within a row, different superscripts indicate significant differences between treatments (*P* < 0.05).

1 Pasture height = low (L), 10 cm, and high (H), 14 cm; concentrate amount = 3, 5, or 7 kg DM of concentrate/cow per day, offered to the cows.

2 SED = standard error of the difference.

3 Pasture utilization calculated using the equation by Langworthy et al. (2021).

As reported by Stockdale (2000), the amount of pasture DMI decreases as the amount of supplementary feed consumed increases. In our experiment, cows offered 7 kg DM of concentrate consumed less pasture regardless of pregrazing pasture height compared with cows offered 3 or 5 kg DM of concentrate. Substitution will affect the marginal milk response, and while profitable concentrate feeding occurs, it can be affected by the price of the concentrate, milk price, and the response to the concentrate (Poole et al., 2019). As pasture is generally the lowest-cost feed source on pasture-based farms, substituting pasture consumption for concentrate consumption can reduce profit (Stockdale, 2000; Heard et al., 2017). Wales et al. (1999) reported that the best use of supplements occurs when pastures are shorter in height (up to 3,100 kg DM/ha pasture mass). The pregrazing pasture masses in the experiment were above the suggested threshold of Wales et al. (1999), and as concentrate amount was part of the experimental design, we could only observe how pasture and total DMI were affected. Pasture intakes were greater for cows offered either 3 or 5 kg DM of concentrate, regardless of the pregrazing pasture height.

Pregrazing mass did not differ between the H treatments; however, there was some unexpected variation in the L treatment. Cows on the L3 treatment were offered pasture of similar pregrazing pasture mass as the H3, H5, and H7 cows. However, pasture quality (presented earlier) did not differ greatly between the treatments. Pasture ME, ADF, and NDF were similar for the L and H treatments, and CP concentration was 17.4% versus 18.7% for L and H, respectively. While postgrazing pasture mass was generally higher for the cows offered 7 kg DM of concentrate per day on both the L and H treatments, this was expected due to the known effects of increased concentrate amount on substitution. Pasture utilization, therefore, was unlikely to be affected by the nutritive characteristics of the pasture and more likely related to pasture mass and the amount of concentrate being offered to the cows in this experiment. Additionally, the similarities in pregrazing pasture mass and nutrient composition of the pasture could have influenced the lack of difference in milk yield between cows on the L and H treatments.

Cows offered 7 kg DM of concentrate/d produced the most amount of milk, cows offered 5 kg DM were intermediate, and cows offered 3 kg DM produced the least (*P* < 0.001; Table 2). Milk protein yield was greatest for cows offered either 5 or 7 kg DM of concentrate/d (*P* = 0.018), whereas milk fat yield was greater (*P* = 0.033) for cows offered 7 kg DM compared with those offered 3 kg DM. Our findings are consistent with Henriksen et al. (2018) who reported higher milk yields from cows offered up to 6 kg/d. Furthermore, Auldist et al. (2013, 2016) found that yields of milk, milk fat, and milk protein increased with increasing amounts of concentrate fed in the parlor (up to 12.5 kg DM/cow per d) during milking in combination with a restricted pasture allowance (nominally 14 kg DM/cow per d). A review by Hills et al. (2015) also found that milk yields over a whole lactation increased when cows were fed increased amounts of concentrate, up to 7.5 kg DM/cow per d.

**Table 2:** Average yields (kg/cow per d) of milk and ECM; milk fat, protein, and lactose yields (kg/cow per d); milk fat, protein, and lactose concentration (%); and BW (kg) of cows in each of the treatments, averaged over the two 6-d measurement periods

Variable	Pasture height^1^		Concentrate amount^1^			Interaction (Int.)						SED^2^			*P*-value		
	L	H	3	5	7	L3	L5	L7	H3	H5	H7	Height	Concentrate	Int.	Height	Concentrate	Int.
Milk yield	18.7	19.1	16.8^a^	19.1^b^	20.8^c^	16.3	19.2	20.6	17.3	19.1	21.0	0.62	0.77	1.08	0.536	<0.001	0.764
ECM yield	20.3	21.0	18.7^a^	21.0^b^	22.2^b^	18.0	21.0	21.8	19.4	21.0	22.5	0.72	0.90	1.26	0.341	<0.001	0.738
Milk protein yield	0.80	0.82	0.72^a^	0.83^b^	0.86^b^	0.72	0.83	0.84	0.76	0.83	0.87	0.033	0.041	0.056	0.528	0.018	0.849
Milk protein percent	3.98	3.92	4.02	3.97	3.87	4.08	4.00	3.87	3.95	3.94	3.88	0.075	0.093	0.131	0.447	0.304	0.787
Milk fat yield	0.81	0.86	0.77^a^	0.85^ab^	0.88^b^	0.73	0.85	0.86	0.81	0.86	0.90	0.035	0.043	0.061	0.223	0.033	0.734
Milk fat percent	4.40	4.51	4.60	4.49	4.27	4.52	4.48	4.21	4.68	4.50	4.33	0.121	0.150	0.211	0.401	0.081	0.889
Milk lactose yield	0.87	0.89	0.77^a^	0.89^b^	0.98^c^	0.75	0.89	0.97	0.79	0.88	0.99	0.030	0.037	0.052	0.617	<0.001	0.784
Milk lactose percent	4.63	4.60	4.57^a^	4.58^a^	4.69^b^	4.58	4.62	4.69	4.55	4.54	4.70	0.039	0.049	0.068	0.396	0.022	0.668
BW	635	637	630	642	638	628	641	637	632	643	638	5.3	6.6	9.2	0.707	0.209	0.987

a–c Within a row, different superscripts indicate significant differences between treatments (*P* < 0.05).

1 Pasture height = low (L), 10 cm, and high (H), 14 cm; concentrate amount = 3, 5, or 7 kg DM of concentrate/cow per day, offered to the cows.

2 SED = standard error of the difference.

Feeding increased amounts of concentrate to cows has also been found to increase milk production by increasing DMI (Auldist et al., 2016). However, in our experiment pasture DMI declined (*P* < 0.001) for cows offered the highest amount of concentrate (7 kg DM; main effect, Table 1). Pasture DMI was, on average, 4 kg DM/cow per d greater for cows offered 3 and 5 kg DM of concentrate/d compared with those offered 7 kg DM. Pasture DMI varied between the 6 treatment groups, as shown in Table 1. Whereas pasture intake differed between cows fed different amounts of concentrate on the H pasture height, total DMI was not significantly different for those cows; however, cows offered 5 kg DM/cow per day on the L pasture height had greater total DMI compared with cows offered 3 or 7 kg DM/d of concentrate (*P* = 0.023). Results from our experiment show that feeding higher amounts of concentrate increases substitution of pasture for concentrate. This is consistent with previous research (Stockdale, 2000; Poole et al., 2019) and is not conducive to farm profitability.

The current experiment found that feeding behavior was not influenced by pregrazing pasture height. There was no difference in total daily eating time between any of the 6 treatment groups (Table 3). The number of graze bites taken per day was greater for cows offered 3 kg DM of concentrate/d compared with those offered 7 kg DM (*P* = 0.034); cows offered 3 kg DM of concentrate took 3,521 more graze bites per day. When reallocating the amount of grain based on milking order for grazing dairy cows, Douglas et al., unpublished (M. D. Douglas, M. M. Wright, V. M. Russo [Agriculture Victoria Research, Ellinbank, VIC, Australia], G. L. Morris, J. W. Heard, A. L. Thomson [Agriculture Victoria Research, Ellinbank, VIC, Australia], M. J. Berkhout, K. Giri, W. J. Wales, and M. J. Auldist [Agriculture Victoria Research, Ellinbank, VIC, Australia]) found that cows offered 4 kg DM of concentrate (compared with 6 and 8 kg DM) consumed more pasture; there was a trend for a greater number of chews per day, but no difference in milk production. Therefore, the increase in graze bites is associated with increased pasture intake, particularly at lower levels of concentrate intake. The similarities in pregrazing pasture mass among some treatments may have contributed to the absence of differences in feeding behavior associated with pasture height. Future research incorporating a greater separation in pasture height treatments and corresponding pasture masses may help elucidate potential effects on feeding behavior.

**Table 3:** Grazing behavior metrics, including time (min/d) spent eating and ruminating, and the number (number/d) of chews and bites taken over a day for cows in each of the treatments, averaged over the two 6-d measurement periods

Variable	Pasture height^1^		Concentrate amount^1^			Interaction (Int.)						SED^2^			*P*-value		
	L	H	3	5	7	L3	L5	L7	H3	H5	H7	Height	Concentrate	Int.	Height	Concentrate	Int.
Eating time	515	522	504	537	514	490	533	521	518	540	507	13.1	16.4	22.9	0.598	0.136	0.436
Graze bites	27,136	26,531	28,692^b^	26,635^ab^	25,174^a^	28,167	26,887	26,354	29,216	26,383	23,994	1,050	1,317	1,842	0.581	0.034	0.425
Eat up^3^ chews	4,957	5,570	4,116^a^	5,436^ab^	6,239^b^	3,719	5,258	5,894	4,514	5,614	6,583	583	730	1,022	0.305	0.017	0.951
Eat down^3^ chews	31,268	30,912	32,276	31,465	29,528	31,721	31,407	30,675	32,831	31,523	28,380	1,165	1,461	2,043	0.772	0.163	0.482
Ruminating time	404	413	419^b^	422^b^	385^a^	412	421	378	425	423	392	11.3	14.2	19.9	0.382	0.019	0.885
Ruminating chews	23,811	24,570	24,684	24,724	23,162	23,757	24,705	22,970	25,612	24,743	23,354	897	1,124	1,572	0.398	0.287	0.681

a,b Within a row, different superscripts indicate significant differences between treatments (*P* < 0.05).

1 Pasture height = low (L), 10 cm, and high (H), 14 cm; concentrate amount = 3, 5, or 7 kg DM of concentrate/cow per day, offered to the cows.

2 SED = standard error of the difference.

3 Eat up and eat down chews: chews made with the cow's head either up or down, respectively.

This experiment aimed to investigate different pregrazing pasture heights and concentrate amounts on milk production, individual cow pasture DMI, and grazing behavior of late-lactation grazing dairy cows. As most farmers in temperate regions of the world use a pasture-based system, ensuring that farmers provide adequate feed that can be well utilized is vital, and the optimal combination of pasture and supplementary concentrate amounts is critical for profitability. Pregrazing pasture height did not influence milk yield and composition; therefore, the first part of hypothesis 1 is rejected. It is likely that the differences in pregrazing pasture height were not great enough between the L and H treatments to result in any significant differences, particularly when considering the pregrazing pasture mass offered to the different treatments. Cows offered 7 kg DM of concentrate produced greater amounts of milk compared with cows offered 3 or 5 kg DM, so the first part of hypothesis 2 is accepted. Pasture DMI was greater for cows offered a higher pregrazing pasture height; however, it declined when fed in combination with an intake of 7 kg DM of concentrate, supporting the second parts of both hypothesis 1 and 2 that pasture DMI increased with pasture height but not concentrate amount. Total daily eating time was not different for cows offered either of the 2 pregrazing pasture heights or 3 concentrate amounts; therefore, the third hypothesis is rejected.

In conclusion, milk yield increased as the amount of concentrate offered increased, but pregrazing pasture height did not influence milk production in this experiment. This lack of significant response may be attributed to the similarity in pregrazing pasture mass across treatments. It is likely that pasture mass, rather than pasture height per se, exerts a greater influence on milk production through its effects on pasture nutritive value and pasture intake. We acknowledge this as a limitation of the present experiment. Future research should examine a wider range of pregrazing pasture heights that correspond to clearly differentiated pasture masses, to better elucidate any potential effects of pasture height on milk production and grazing behavior, and should also investigate botanical composition over different pregrazing pasture heights. Pasture intake is negatively affected when feeding greater than 5 kg DM of concentrate/d in late lactation, typically resulting in an increased postgrazing residual. Offering pasture at a lower pasture mass, which is typical of a lower pregrazing pasture height, will ensure more efficient pasture utilization. Finding the balance between concentrate amount and efficient pasture utilization at different stages of lactation is, although difficult, a central principle in managing a profitable pasture-based dairy farm in temperate dairy regions. Milk yield responses observed here would be expected to be different at other stages of lactation. Additionally, variation in the nutritive characteristics of the pasture at different growth stages (e.g., vegetative vs. reproductive) in different seasons may exhibit different milk yield responses; further research would be required at different times of the year and stage of lactation to elucidate this.
